# Post-traumatic stress syndromes following childbirth influenced by birth mode—is an emergency cesarean section worst?

**DOI:** 10.1007/s00404-023-07114-5

**Published:** 2023-07-01

**Authors:** Beate Hüner, Thomas Friedl, Sabine Schütze, Arkadius Polasik, Wolfgang Janni, Frank Reister

**Affiliations:** Department for Gynecology and Obstetrics, University Hospital Ulm, Prittwitzstraße 43, 89075 Ulm, Germany

**Keywords:** Post-traumatic stress syndromes, Emergency cesarean section, City birth trauma scale, IES-R, Debriefing

## Abstract

**Purpose:**

The experience of birth is an emotional challenge for women. Traumatic birth experiences can cause psychological stress symptoms up to post-traumatic stress disorders (PTSD), with impact on women’s wellbeing. Primarily unplanned interventions can trigger birth-mode-related traumatization. The aim of the study was to evaluate whether an emergency cesarean section (ECS) is the most traumatizing.

**Methods:**

A retrospective case–control study was undertaken. Therefore, data were collected by standardized questionnaires (Impact of Event Scale-Revised and City Birth Trauma Scale) that were sent to women with singleton pregnancies > 34 weeks of gestation who either give birth by ECS (case group, *n* = 139), unplanned cesarean section (UCS), operative vaginal birth (OVB), or natural birth (NB) (three control groups, *n* = 139 each). The investigation period was 5 years.

**Results:**

Overall, 126 of 556 (22%) sent questionnaires were returned and could be analyzed (32 ECS, 38 UCS, 36 OVB, and 20 NB). In comparison to other birth modes, women with ECS were associated with a higher degree of traumatization as revealed by statistically significant differences regarding the DSM-5 criteria intrusion and stressor. In addition, women who underwent ECS declared more frequently a demand for professional debriefing compared to other birth modes.

**Discussion:**

ECS is associated with more post-traumatic stress symptoms compared to other birth modes. Therefore, early interventions are recommended to reduce long-term psychological stress reactions. In addition, outpatient follow-ups by midwives or emotional support programs should be implemented as an integral component of postpartum debriefings.

## What does this study add to the clinical work?

**Table:** 

The results of the study create awareness for birth-mode-related traumatization after unexpected intervention during birth. Therefore, debriefing after traumatic birth experiences should be implemented in clinical work in obstetric care.	

## Introduction

The experience of birth is an emotional challenge. If unexpected interventions are necessary, the expectant mother is afraid for her own life or that of the unborn child. This can lead to emotional stress reaction up to childbirth-related post-traumatic stress disorder (CB-PTSD) [1, 2]. About 30–44% of women experience a stressful up to a traumatic childbirth and 1–3% develop a CB-PTSD [3]. As a trigger for a PTSD, *birth* was already included in the Diagnostic and Statistical Manual of Mental Disorders (DSM-5) [4] in 1994 [5]. PTSD was characterized by the presence of trauma exposure with symptom presence for a month with at least one of the following symptoms: intrusion, avoidance, negative mood and cognitive changes as well as hyperarousal and reactivation [6]. CB-PTSD due to birth trauma can affect maternal wellbeing as well as mother–child relationship and the partner relationship [7]. However, it is not regularly assessed in the postpartum routine care [8]. Trigger of traumatic birth experiences include labor pain, anxiety before childbirth, concern for the newborn child, pre-existing depression, and lack of support during childbirth. Another important factor seems to be the birth mode and unplanned interventions in emergency situations. In particular, emergency cesarean section (ECS) but also operative vaginal birth (OVB) may be responsible for the development of CB-PTSD [9, 10]. Unfortunately, the diagnosis of a CB-PTSD is often inconclusive and easily missed [11]. For evaluation, there are a variety of standardized and validated questionnaires that allow the diagnosis of CB-PTSD, including the Impact of Event Scale-Revised (IES-R) [12, 13]. Only few questionnaires are specifically designed for the implementation in an obstetric setting [14, 15]. A questionnaire to measure CB-PTSD was developed by The City Birth Trauma Scale (CBiTS), a 29-item questionnaire [16]. It is the first diagnostic tool for CB-PTSD according to the DSM-5 criteria and the only validated questionnaire for CB-PTSD in Germany [8].

The aim of the present study is to investigate the association of childbirth-related traumatization depending on different birth mode using a retrospective survey in a long period from 17 to 89 month postpartum. Using a case–control design, the study aims to answer the question of whether an emergency cesarean section (ECS) is the most traumatic birth mode. Additionally, the study evaluates the request and timing for postpartum debriefing.

## Materials and methods

### Study design and cohort

To measure the degree of traumatization, the CBiTS and IES-R were used for a retrospective survey. The diagnosis of PTSD was not part of the research question. To measure and compare birth-mode-related traumatization assessed based on the responses to the CBiTS and IES-R questionnaires, a case–control study was designed under the hypothesis an ECS leads to a stronger traumatization compared to other birth mode. Therefore, a case group with 139 women (ECS) was compared to 3 control groups (UCS, OVB, and NB), each with 139 women, in summary 556 participants. Data were recorded at the Department of Gynecology and Obstetrics of the University Hospital Ulm, an obstetric led care prenatal center level I from the years 2014 to 2019. Using the Operation and Procedure Classification System (OPS-Code), all emergency cesarean sections were identified in the database. In addition, these files were reviewed individually according to the inclusion criteria. Multiple births, elective cesarean sections, and premature births < 34 weeks of gestation were excluded. To reduce the impact of uncontrollable factors (e.g., different healthcare team, different obstetric management), for each ECS, the timely nearest birth in the same setting was selected for the control groups. The participants received the questionnaires by postal mail. Unfortunately, for the study, only postal contact was made for the survey without prior written information or personal contact due to resource constraints. The births occurred up to 5 years ago, and updating the address was not possible. As a result, many letters could not be delivered. Consent to the study was confirmed by the signed and returned letter of consent. The study was conducted in accordance with the Declaration of Helsinki, and the protocol was approved by the Ethics Committee of the University Ulm under application number 184/20.

### Data collection

The IES-R questionnaire comprises 22 questions to evaluate the expression or frequency of feelings about a stressful incident (here: birth). For this study, a validated translated German version of the questionnaire [13, 17] was used with a four-level rating scale. The questions are structured along three subscales “intrusion”, “avoidance”, and “hyperarousal” according to the DSM-5 criteria. The total score of each sub-scale can be subjected to statistical analysis, allowing for comparisons based on the birth mode.

The CBiTS questionnaire is the first specifically developed questionnaire to investigate CB-PTSD-related symptoms. It covers all DSM-5 criteria for PTSD, “stressor”, “intrusion”, “avoidance”, “negative mood and cognitions”, and “hyperarousal”. Stressor criteria are queried on a two-stage evaluation scale “no” and “yes”. For the remaining DSM-5 criteria, a four-level rating scale is used. Furthermore, symptoms onset, duration, and impact are queried. For statistical analysis, response scales are treated as categorical (stressor criteria, symptoms onset, duration, and impact) or ordinal scales (other criteria).

In addition, further questions were posed regarding the occurrence or need for postnatal debriefings, either during the hospital stay or 3–6 months after birth, with the involvement of the midwife, doctor present at birth, another doctor, and/or a psychologist. Originally, the CBiTS framework also comprised such questions; however, they were excluded in the available versions [16].

### Statistical analysis

The IES-R results for the four birth mode groups in terms of the total scores for each of the three subscales were initially compared among all birth modes using the Kruskal–Wallis test. We used the non-parametric test due to the ordinal rating scale of the questions. In case of a significant result of the Kruskal–Wallis test (*p* < 0.05), we performed post hoc pairwise comparisons with p values adjusted for multiple comparisons using Bonferroni correction. CBiTS results for the questions regarding the criteria “intrusion”, “avoidance”, “negative mood and cognitions”, and “hyperarousal” with ordinal scale response categories, were analyzed in the same manner. The two stressor questions with only “yes” or “no” as possible answers as well as the questions regarding onset, duration, and impact of symptoms and the questions regarding debriefing were analyzed using the Chi-square test.

We used Python programming language (version 3.9.5) with NumPy (version 1.21.0) and pandas (version 1.2.5) libraries for data processing, SciPy library (version 1.7.0) for statistical analysis, and Matplotlib library (version 3.4.2) for plotting information graphics. All statistical tests were two sided.

## Results

### Baseline characteristics

One hundred and twenty-six questionnaires were returned after 3 months and could be evaluated (response rate 22%). The sample comprised 32 (23%) ECS, 38 (27%) UCS, 36 (25%) OVB, and 20 (14%) NB. Table 1 describes the baseline characteristics of the participants. Most women were primiparous, median age was 33, and the time between childbirth and survey ranged between 17 and 89 months.

**Table 1:** Baseline characteristics

Baseline characteristics		Overall	ECS	UCS	OVB	NB
Sample	Participants	556	139	139	139	139
	Responses	126	32	38	36	20
	Return rate	22%	23%	27%	26%	14%
Age mother (years)	Median	33.0	32.0	35.0	31.0	32.5
	Interquartile	11.00	5.50	7.00	5.25	3.25
	Min	25	26	26	25	26
	Max	43	43	43	40	39
Parity	Primiparous	90	24	26	29	11
	Multiparous	36	8	12	7	9
Age child (months)	Median	42.0	41.5	35.0	47.5	52.5
	Interquartile	36.00	19.75	29.25	34.25	28.25
	Min	17	18	17	17	20
	Max	89	89	88	87	85

### Evaluation of questionnaires

#### Impact of event scale-revised

**Fig. 1:**
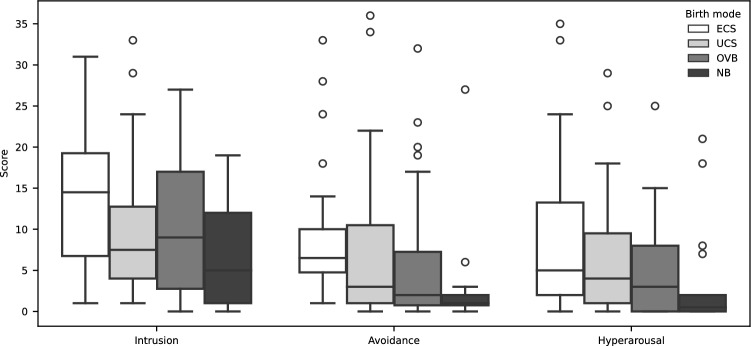
Degree of traumatization per birth mode according to IES-R questionnaire (circles represent outliers, see Tables 2, 3 for significance analysis and *p* values)

The IES-R questionnaire captures three typical forms of reactions to potentially stressful events: intrusion, avoidance, and hyperarousal. The overall comparison of the total scores of these three items revealed statistically significant differences among the four birth modes (Table 2). Pairwise comparisons between birth modes show significant differences between ECS and NB regarding intrusion, avoidance, and hyperarousal, and between ECS and OVB regarding avoidance. No other statistically significant differences between birth modes could be detected (see Fig. 1 and Table 3).

**Table 2:** IES-R analysis, *p* values by Kruskal–Wallis test for overall comparisons of the score sum of each sub-scale among the four birth modes, *< 0.05, **< 0.01, ***< 0.005, ****< 0.001

Item	Overall
Intrusion	0.006**
Avoidance	< 0.001****
Hyperarousal	0.021*

**Table 3:** IES-R analysis, *p* values for pairwise comparisons of IES-R subscales between birth modes after significant Kruskal–Wallis test (see Table 2), *p* values adjusted for multiple comparisons (Bonferroni correction), *< 0.05, **< 0.01, ***< 0.005, ****< 0.001

Item	ECS / UCS	ECS / OVB	ECS / NB	UCS / OVB	UCS / NB	OVB / NB
Intrusion	0.072	0.168	0.006**	1.000	1.000	0.853
Avoidance	0.117	0.009**	< 0.001****	1.000	0.066	0.462
Hyperarousal	1.000	0.350	0.019*	1.000	0.162	1.000

Summarizing the IES-R results, the degree of traumatization of women with an ECS measured with the psychological DSM-5 response criteria—intrusion, avoidance, and hyperarousal—is significantly higher compared to NB and compared to OVB significantly higher in the criteria avoidance.

### City birth trauma scale

The CBiTS questionnaire includes the DSM-5 criteria—stressor, intrusions, avoidance, negative mood and cognitions, and hyperarousal.

Table 4 provides the overall comparisons of all CBiTS items. Significant differences among the four birth modes were found regarding Stressor 1, 2, Intrusion 4, 5, Negative Mood and Cognition 4 and Hyperarousal 5 and Impact 2. Pairwise comparisons following significant overall tests (Tables 5, 6) showed a statistically significant difference for intrusion between ECS and NB for items Intrusion 4 (*Getting upset when reminded of the birth*) and Intrusion 5 (*Feeling tense or anxious when reminded of the birth*). Furthermore, a statistically significant difference was found between ECS and NB for item Negative Mood and Cognition 4 (*Feeling negative about myself or thinking something awful will happen*). While pairwise comparisons revealed no statistically significant difference between birth modes with regard to Stressor 1 (*Did you believe you or your baby would be seriously injured?*), and Impact 2 (*Do they prevent you doing things you usually do, e.g., socializing, daily activities*?), Stressor 2 (*Did you believe you or your baby would die?*) differed significantly between ECS and each of the other three birth modes (Table 5), with women in the ECS group, a significantly higher number, believed that they or their baby would die compared to UCS, OVB, and NB (69%, 16%, 31%, and 5%, respectively).

**Table 4:** CBiTS analysis, *p* values by Chi-squared test (stressor criteria, symptoms onset, duration, and impact) and Kruskal–Wallis test (other criteria) for overall comparisons among the four birth modes, *< 0.05, **< 0.01, ***< 0.005, ****< 0.001

Item	Overall
Stressor 1	0.013*
Stressor 2	< 0.001****
Intrusions 1	0.326
Intrusions 2	0.597
Intrusions 3	0.254
Intrusions 4	0.004***
Intrusions 5	0.007**
Avoidance 1	0.748
Avoidance 2	0.540
Negative M&C 1	0.069
Negative M&C 2	0.300
Negative M&C 3	0.154
Negative M&C 4	0.016*
Negative M&C 5	0.222
Negative M&C 6	0.252
Negative M&C 7	0.927
Hyperarousal 1	0.621
Hyperarousal 2	0.371
Hyperarousal 3	0.813
Hyperarousal 4	0.652
Hyperarousal 5	0.042*
Hyperarousal 6	0.554
Hyperarousal 7	0.514
Hyperarousal 8	0.916
Onset	0.232
Duration	0.075
Impact 1	0.389
Impact 2	0.032*
Impact 3	0.241

**Table 5:** CBiTS analysis, *p* values for pairwise comparisons of CBiTS criteria between birth modes after significant Chi-squared test (see Table 4, *p* values adjusted for multiple comparisons (Bonferroni correction), *< 0.05, **< 0.01, ***< 0.005, ****< 0.001

Item	ECS / UCS	ECS / OVB	ECS / NB	UCS / OVB	UCS / NB	OVB / NB
Stressor 1	0.238	6.0000	0.238	0.290	5.015	0.283
Stressor 2	< 0.001****	0.022*	< 0.001****	1.306	2.630	0.350
Impact 2	0.496	4.972	3.625	0.271	0.420	4.493

**Table 6:** CBiTS analysis, *p* values for pairwise comparisons of CBiTS criteria between birth modes after significant Kruskal–Wallis test (see Table 4, *p* values adjusted for multiple comparisons (Bonferroni correction), *< 0.05, **< 0.01, ***< 0.005, ****< 0.001

Item	ECS / UCS	ECS / OVB	ECS / NB	UCS / OVB	UCS / NB	OVB / NB
Intrusions 4	0.391	0.207	0.002***	4.891	0.250	0.337
Intrusions 5	2.243	0.145	0.013*	1.056	0.078	0.594
Negative M&C 4	2.551	0.445	0.015*	1.885	0.072	0.346
Hyperarousal 5	0.638	5.381	0.919	0.510	0.074	1.171

If symptoms existed, onset, duration, and impact of the symptoms were queried. Symptoms occurred mostly in the first 6 months and most often lasted longer than 3 months. Overall comparisons revealed no significant difference among the four different birth modes regarding onset or duration of symptoms, Impact 1, or Impact 3 (Table 4). While there was a significant difference of Impact 2 (*Do they prevent you doing things you usually do (e.g., socializing, daily activities)?*) in the overall comparisons among birth mode groups, the (Bonferroni-corrected) pairwise comparisons yielded no significant result.

### Evaluation of request for postnatal debriefing depending on birth mode

The overall comparisons of the responses to the debriefing questions revealed significant differences in the frequencies of response categories among birth modes regarding Debriefing 1 (*p* < 0.001) and Debriefing 4 (*p* = 0.011), but not about Debriefing 2 (*p* = 0.134) or Debriefing 3 (*p* = 0.184). Bonferroni-corrected pairwise comparisons for Debriefing 1 (*I had a debriefing in the hospital about the birth process*) showed significant differences in response frequencies between ECS and UCS (*p* = 0.003), ECS and OVB (*p* = 0.002), and ECS and NB (*p* = 0.004), with most debriefings with the doctor present at birth or another doctor in the ECS group. For Debriefing 4 (*I would have liked to debrief the birth process after 3–6 months*), the only significant difference obtained by pairwise comparisons after Bonferroni correction was found between ECS and UCS (*p* = 0.039). Women in the ECS group more frequently had the wish to discuss the birth process with the doctor present at birth and with a psychologist after 3 to 6 months compared to women in the UCS group (34% vs. 15% and 26% vs. 7%, respectively).

## Discussion

The results of the standardized questionnaires reveal a stronger traumatization by ECS compared to the other birth modes and confirm the hypothesis ECS is worst. Especially in the criteria intrusion and negative mood and cognition, the ECS shows a statistically significant higher degree of traumatization compared to the other birth modes. These reactions are characterized by negative memories of the birth process as well as negative feelings toward oneself. This is in line with existing studies. Women who give birth by cesarean section show more frequent a feeling of disappointment, poor body feeling, and lower self-confidence. This could be a long-term risk for developing a depression [14, 15]. Similar results with significant undesirable psychological effects depending on birth mode are found in a study with 272 women. A worsening of mood and a decrease in self-esteem could be detected in women who give birth by cesarean section [18]. A study with 685 women also showed that birth mode has an impact on maternal mental health and postpartum development of CB-PTSD [19]. A higher risk of mental health problems after unplanned cesarean sections was observed. In addition, the need for postpartum interventions after unplanned cesarean sections is highlighted to reduce the risk of developing CB-PTSD [19]. In a large survey study from England, more than 5,300 women were asked about symptoms of CB-PTSD, anxiety, and depression 3 months after birth [20]. The results also showed a higher risk for CB-PTSD if an UCS or OVB was necessary; however, there was no distinction between UCS and ECS [20]. In contrast, a large Norwegian study found no association between birth mode (with a distinction made between planned cesarean section and ECS) and postpartum emotional stress, measured with a short form of the Hopkins symptom checklist-25 (SCL-8) at 30 weeks of gestation and at 6 months postpartum [21].

Other studies focused on possible risk factors for childbirth-related trauma. Here, ECS is identified as a possible risk factor. Identifying risk factors for childbirth-related trauma can enable targeted screening and the use of interventions and preventions to prevent post-traumatic stress reactions [22]. In one study, both so-called prenatal, birth-related, and postnatal social support and cognitive variables are described as risk factors for the development of post-traumatic symptoms. Again, ECS as a birth-related variable showed significantly higher risk for developing a post-traumatic stress response [23]. In two recent literature reviews, the impact of birth mode involving emergency interventions on CB-PTSD were identified both in quantitative and qualitative study designs [24, 25].

In awareness that birth mode can be a risk factor for mental stress response up to CB-PTSD, debriefing interventions could be useful [26]. A systematic review provides an overview of potential secondary interventions for CB-PTSD. One method that has shown a significant impact is expressive writing after the traumatic birth experience [27]. Another approach is an immediate debriefing of the birth-related trauma by a midwife-led counseling intervention [28]. In our collective, this was most often desired by women with ECS. Near-time debriefing of the stressors “Did you believe your baby would be seriously injured?” and “Did you believe you or your baby would die?” can already be done in hospital by a midwife or obstetrician. These questions were most frequent answered with *yes* by women with ECS. In this setting, questions about the process of birth and necessary interventions during birth due to complications could be explained. In addition, outpatient follow-up by a midwife is an important part of postpartum debriefing. But not only an immediate debriefing of a traumatizing birth seems to be useful, but also after a longer time interval. Our results confirm that women with an ECS were more likely to request a debriefing at 3–6 months compared with women with a UCS. Therefore, implementing special midwifery-led emotional support programs can improve outcomes after a traumatic birth experience [26].

### Strength and limitation

Strength of our study is the detailed distinction between four different modes of birth and a survey over a long retrospective period using standardized validated CB-PTSD questionnaires. The additional questioning regarding debriefing interventions which is often not queried in present literature could be used for future improvement in the prevention of developing long-term CB-PTSD.

Limitation of the study is the low number of cases. This is due to the rather low response rate. Unfortunately, in the study design, it was not possible to establish personal contact with the women to obtain their consent and current address. In addition, the long duration of the interview period likely had an impact on participants' willingness to participate and a certain distortion of memories. However, the individual groups were similar in terms of the time elapsed since birth. Therefore, the results of the survey from the individual groups can be compared. For comparison, other studies evaluated a data collection time ranged from 6 months even to 18 years [24]. Furthermore, we cannot rule out the possibility that women already had pre-existing depression or CB-PTSD.

### Future

A targeted screening at various stages during pregnancy to identify potential pre-existing risk factors for a CB-PTSD should be established. Early debriefing interventions and a timely postpartum long-term follow-up with appropriate questionnaires could be implemented especially when pre-existing risk factors or risk factors such as unplanned birth mode are presented. Future research may explore various debriefing intervention methods, preferably through randomized controlled trials.

## Conclusion

An unexpected birth experience can lead to traumatization up to CB-PTSD even with a long-term effect. ECS appears to be associated with the strongest trauma among different birth modes. Therefore, birth mode influences the degree of traumatization and could be regarded as a birth-related risk factor. Identifying women with pre-existing risk factors and birth-related risk factors should be a component in the perinatal care of pregnant women. Special validated questionnaires such as the IES-R or the CBiTS can be used for postpartum evaluation of CB-PTSD-related symptoms. A prevention of possible CB-PTSD and a reduction of long-term traumatization could be achieved with focused interventions for debriefing. Besides an immediate debriefing after a traumatic birth experience, an offer for debriefing should be made after a time interval of 3–6 months. Interventions with trained staff or special midwife-led consultations could be a possible approach.

## Abbreviations

IES-R: Impact of event scale-revised
CBiTS: City birth trauma scale
PTSD: Post-traumatic stress disorder
CB-PTSD: Childbirth-related PTSD
DSM-5: Diagnostic and statistical manual version 5
NB: Natural birth
OVB: Operative vaginal birth
ECS: Emergency cesarean section
UCS: Unplanned cesarean section

## Declarations

### Data availability

The Data is available from the corresponding author on request.
